# Fatal nephrobronchial fistula arising from xanthogranulomatous pyelonephritis: a case report

**DOI:** 10.3389/fmed.2024.1374043

**Published:** 2024-08-14

**Authors:** María Alejandra Burbano, H. A. Nati-Castillo, Natalia Castaño-Giraldo, Gildardo López, Romina Placencia-André, Camila Salazar-Santoliva, Melissa Villavicencio-Gomezjurado, Juan S. Izquierdo-Condoy

**Affiliations:** ^1^Interinstitutional Internal Medicine Group (GIMI 1), Department of Internal Medicine, Universidad Libre, Cali, Colombia; ^2^Urology Department, School of Medicine, Universidad de Cartagena, Cartagena, Colombia; ^3^One Health Research Group, Universidad de las Americas, Quito, Ecuador

**Keywords:** nephrobronchial fistula, lung abscess, chronic infection, xanthogranulomatous pyelonephritis, fatal outcome

## Abstract

**Introduction:**

Nephrobronchial fistula is an exceptionally rare complication of renal infections, including the uncommon xanthogranulomatous pyelonephritis. Existing literature is limited to a few case reports, with antibiotic therapy and nephrectomy being the preferred treatments.

**Case:**

We present the case of a 63-year-old woman with a history of recurrent xanthogranulomatous pyelonephritis in her right kidney, requiring drainage through lumbotomy. She presented with a chronic dry cough and weight loss, without other noticeable symptoms. Imaging suggested a pulmonary abscess and nephrobronchial fistula. Despite antibiotic treatment and surgical intervention, her condition progressed fatally.

**Conclusion:**

Nephrobronchial fistulas are extremely complications of renal infections, often presenting with nonspecific symptoms. This case highlights their significant impact on morbidity and mortality, especially in resource-limited settings, and underscoring the urgent need for prompt diagnosis and treatment.

## 1 Introduction

Fistulas between the kidney and the thoracic cavity are rare events that can arise from multiple causes, including abdominal trauma, complicated lithiasis, and, in very rare cases, renal infections such as tuberculosis, renal abscesses, and xanthogranulomatous pyelonephritis (1–3). Xanthogranulomatous pyelonephritis is a rare, aggressive variant of pyelonephritis, typically related to chronic infection and nephrolithiasis, resulting in a nonfunctioning kidney characterized by the destruction and replacement of renal or perirenal tissue with granulomatous tissue containing lipid-laden macrophages (4, 5).

The first documented case of a nephropulmonary fistula dates back to the 19th century, with approximately 70 cases reported in the first half of the 20th century (6). Since then, the incidence of nephropulmonary fistulas of infectious origin has drastically decreased, primarily due to the availability of antibiotics that control renal infections (7, 8). Currently, nephrobronchial fistulas of infectious origin are exceedingly rare, and the association of xanthogranulomatous pyelonephritis with pulmonary abscess and nephrobronchial fistula is particularly uncommon (2, 9–11).

The standard treatment protocol involves initial antibiotic therapy followed by nephrectomy, which remains the preferred management strategy. Establishing an early diagnosis has proven to reduce further complications (11–13).

In this report, we present a case of xanthogranulomatous pyelonephritis with pulmonary abscess formation and nephrobronchial fistula, diagnosed via computed tomography in a patient with a long-standing cough and ongoing weight loss, which ultimately resulted in a fatal outcome after surgical intervention.

## 2 Case presentation

We present the case of a 63-year-old female patient with a history of biomass exposure, xanthogranulomatous pyelonephritis, and perirenal collection managed with drainage by lumbotomy 3 years ago. She presented to a primary care outpatient clinic with long-standing, nonspecific respiratory symptoms, including a persistent dry cough lasting for 2 years and associated weight loss. Approximately 2 weeks later, an ultrasound revealed a right lung abscess, bronchiectasis in the upper lobe of the left lung, and an abnormal lung-kidney communication, which led to hospital admission.

**Figure 1:**
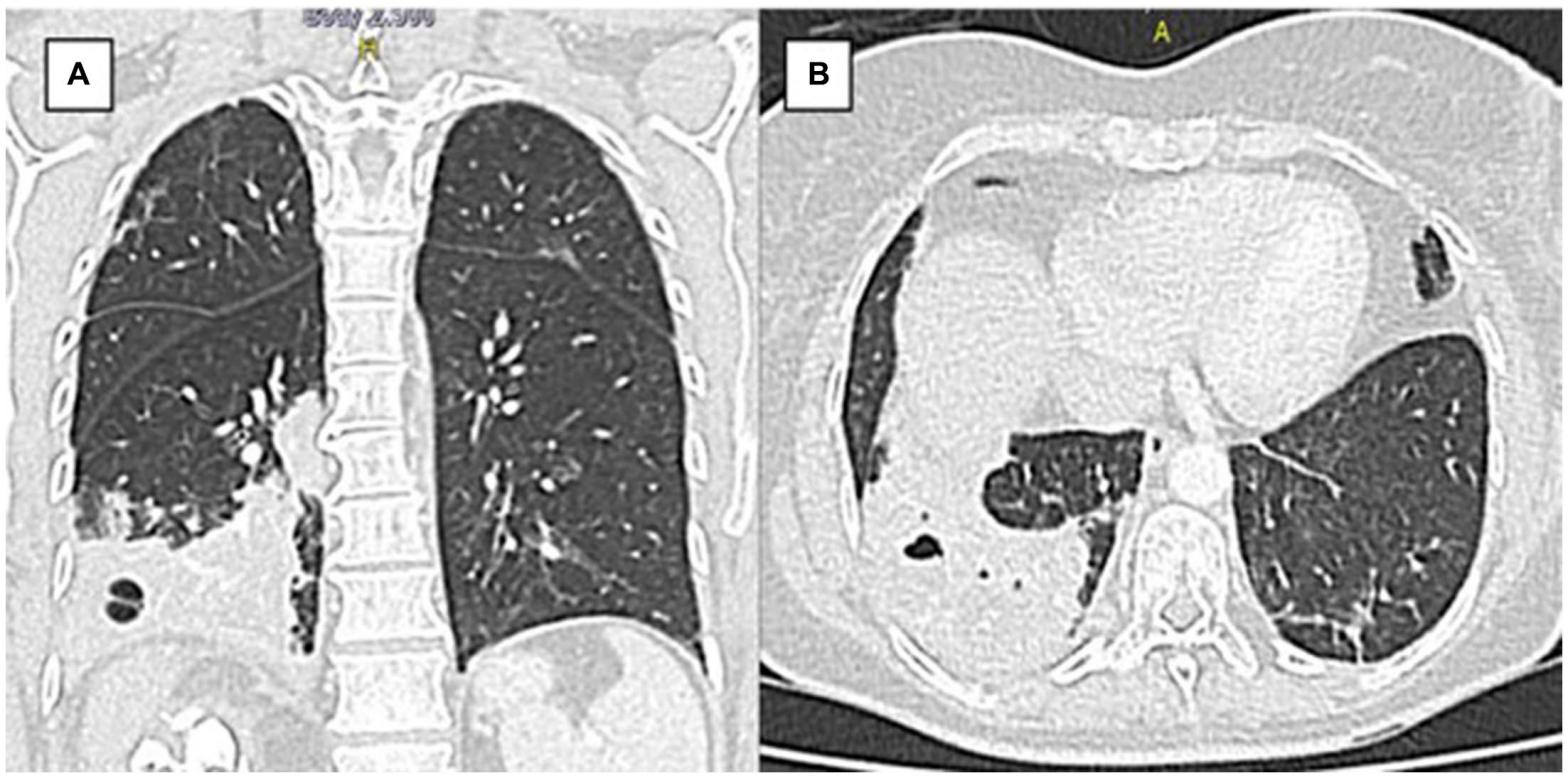
Chest tomography pulmonary **(A)** Coronal section. **(B)** Axial section. Axial view. There is extensive heterogeneous alveolar occupation involving basal segments of the right lower lobe, with intraparenchymal collection with hydroaerial level of 24 mm in diameter.

**Figure 2:**
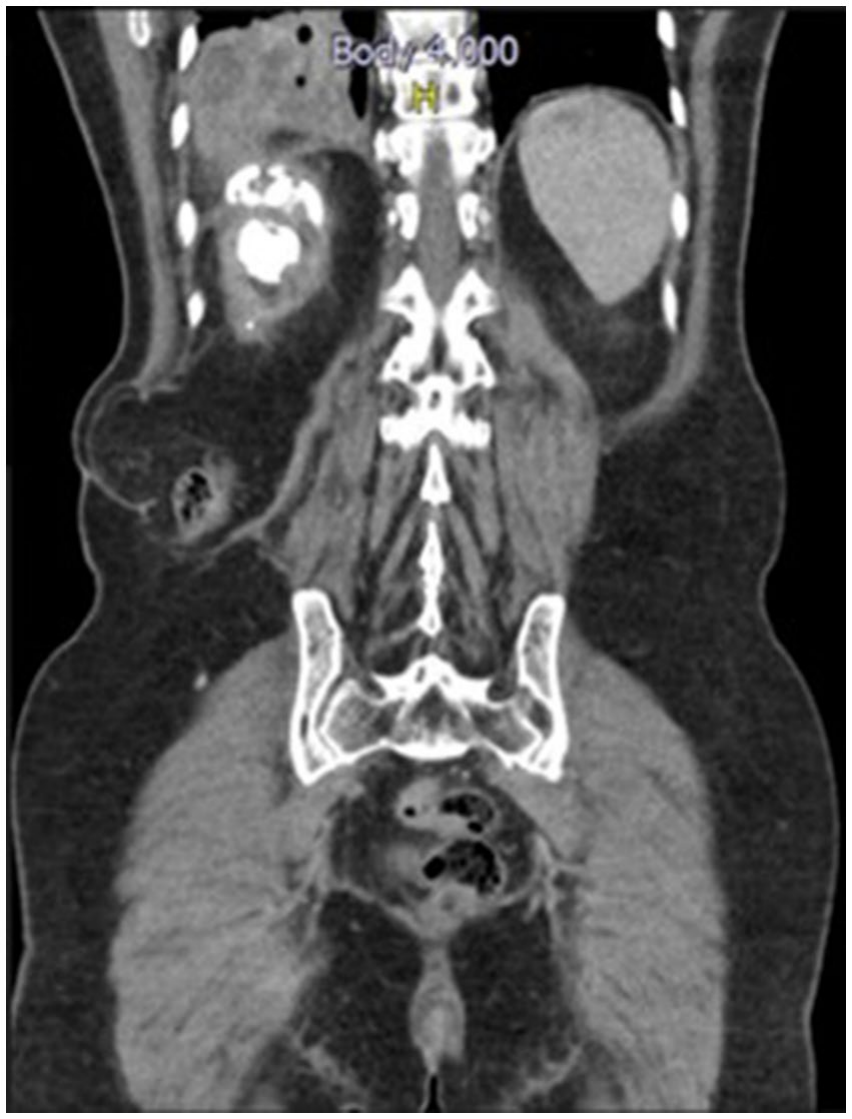
CT scan of the abdomen and pelvis with soft tissue contrast window sagittal view shows a coralliform calculus occupying all the right renal collectors with active fistula between the upper collector and the right basal lung parenchyma.

Seven days later, upon admission, the physical examination revealed an acceptable general condition, with a heart rate and respiratory rate at the upper limit of normal and positive percussion pain in the right flank. Laboratory tests, including a complete blood count and C-reactive protein levels, showed no evidence of a systemic inflammatory response, while urinalysis indicated pyuria without bacteriuria. Additionally, urine and blood cultures showed no microbial growth after 48 h of evaluation. However, contrast-enhanced computed tomography (CT) scan of the thorax and abdomen showed extensive heterogeneous alveolar occupation with a collection and a hydroaerial level (Figure 1). The largest abscessed communicated with the upper collecting system of the ipsilateral kidney, featuring an active fistula approximately 17 mm in transverse diameter and 32 mm in length, containing fluid, air bubbles, and calcifications of about 10 mm. In addition, a coralliform calculus measuring 85 × 34 × 40 mm within the collecting system was noted, thinning the wall and altering the architecture of the genitourinary system (Figure 2). Given the imaging findings, the patient was diagnosed with necrotizing pneumonia in the right lower lobe (RLL), in contact with the renal lesion on the same side. Initial management included empirical antibiotic therapy with ampicillin/sulbactam, which was later switched to piperacillin/tazobactam. Right renal exclusion was confirmed by scintigraphy after 13 days of antibiotic therapy.

During antibiotic management, a staged surgical resolution was planned, starting with a laparoscopic nephrectomy 13 days after admission, followed by a lower lobe lobectomy. During the first surgical stage, lax peritoneal adhesions and a retroperitoneal plastron were encountered, leading to an open right nephrectomy. Extensive inflammatory involvement compromised the base of the right hemidiaphragm, sub-hepatic bed, and vena cava, necessitating repair of the right hemidiaphragm.

In the immediate postoperative period, the patient developed hypovolemic shock, requiring double vasopressor support with norepinephrine and vasopressin at maximum doses, as well as the initiation of inotropic support. She had limited tolerance to the withdrawal of these medications. Hours later, emergency second-stage surgery revealed a 1,500 cc retroperitoneal bleed and bleeding in the adrenal bed with no identifiable vascular injury. Despite aggressive treatment, which included triple vasopressor support and transfusion of 10 units of red blood cells in 24 h, the patient remained hemodynamically unstable.

During this period, the patient developed multiorgan failure, initially presenting with rapidly evolving acute kidney injury requiring hemodialysis. Elevated bilirubin levels, progressive respiratory compromise, and severe metabolic acidosis were identified and treated with insulin and bicarbonate infusion. In addition, the patient developed bleeding from the surgical wound, as well as at the nasogastric and vaginal sites, leading to a decrease in hemoglobin levels to 4 g/dL. In this state, the patient showed a slight prolongation in laboratory coagulation times and international normalized ratio (INR). The platelet count was at the lower limit of normal, and there was no evidence of fibrinogen consumption, thus not meeting the criteria for disseminated intravascular coagulation.

**Figure 3:**
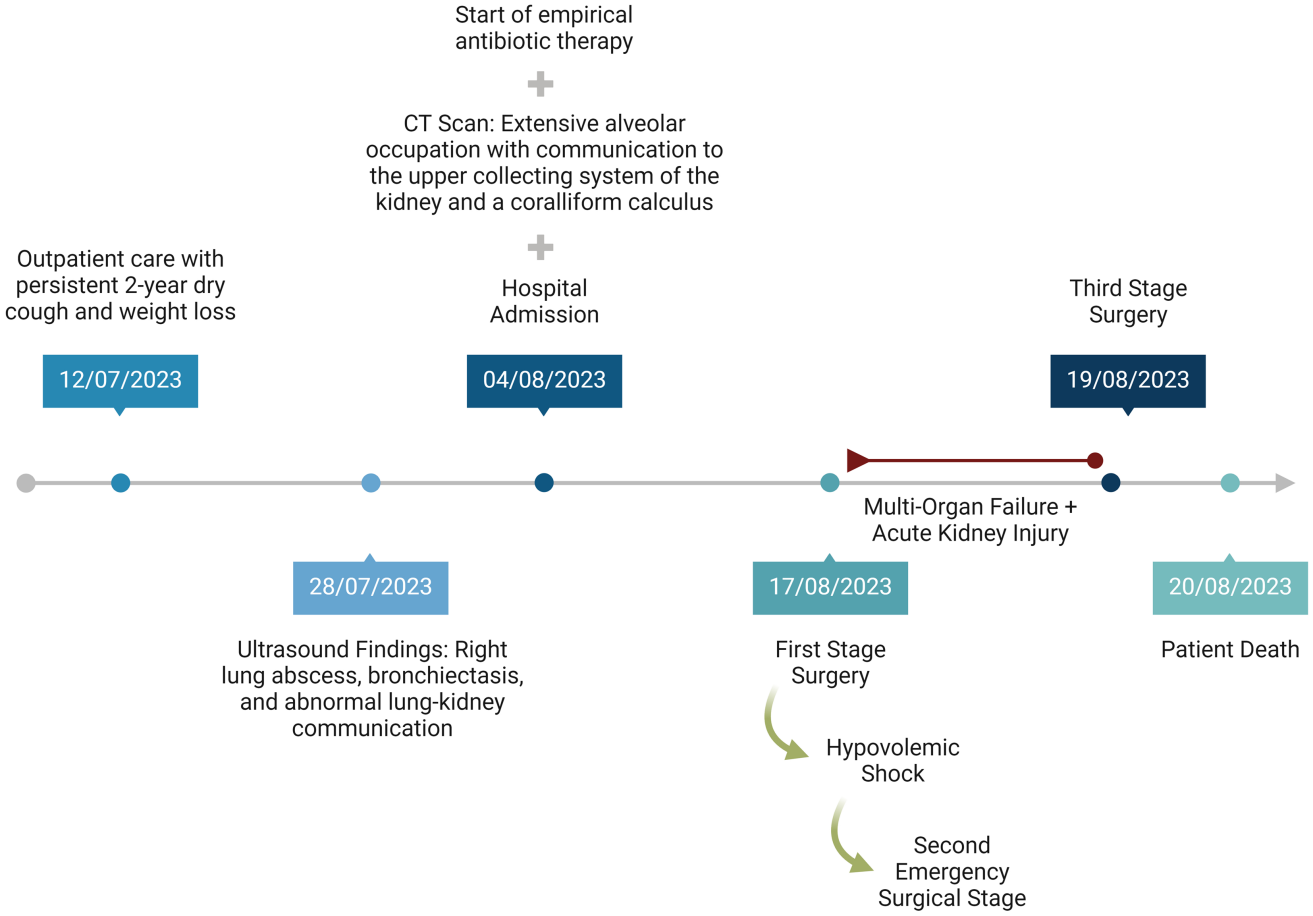
Timeline of patient management.

A third surgical intervention revealed a new collection of serohematic fluid and patchy ischemia in the small and large bowel, leading to concerns about generalized intestinal ischemia. The renal fossa was repacked, and the abdomen was left open with VAC therapy. Despite these efforts, persistent hemodynamic instability led to the patient’s demise. Figure 3 shows the chronology of patient events.

The final pathological report of the right kidney showed severe acute and chronic pyelonephritis with intrarenal abscess and terminal changes due to hydronephrosis caused by nephrolithiasis. Dystrophic calcifications with bone metaplasia were also present in the ureter, indicating slight chronic involvement, with no perirenal or vascular compromise.

## 3 Discussion

In this case, chronic xanthogranulomatous pyelonephritis was a risk factor that precipitated the subsequent chain of events. This condition, being exceedingly rare and aggressive, accounts for merely 1% of all renal infections. It is associated with nephrolithiasis and persistent infection, leading to the replacement of renal and perirenal tissues with extensive granulomatous tissue and infiltration by fatty macrophages. This process distorts the renal architecture and ultimately results in a non-functional kidney, as observed in this case (14).

The etiology of this condition typically involves microorganisms like those causing urinary tract infections in the general population, often facilitated by obstructive processes such as lithiasis. In 80% of these cases, coraliform calculi are observed, as in this patient (7). The presence of abscesses and fistulas as complications, including those with different outlets, is not unusual, presenting diagnostic challenges due to the largely nonspecific symptomatology dependent on the affected tissues. Symptoms may range from general malaise, fever, and weight loss to more specific symptoms like flank or abdominal pain, dry or productive cough, foul-smelling sputum, hemoptysis, uroptisis, and a taste of urine in the mouth (15, 16). According to the findings of the review by Tamburrini et al. (7), from 21 nephrobronchial fistula cases, respiratory symptoms were the most common (47.6%), with productive cough present in half of the cases, and urinary symptoms in 42.9%, differing from this case which presented with only a dry cough and weight loss.

Despite the low incidence of nephrobronchial fistulas since the latter half of the 20th century—with only 31 cases reported since 1949 (7). Nephrobronchial fistulas are the most common type of reno-visceral fistulas, followed by nephrocolonic fistulas, and can pose serious risks to patient survival (13). This case exhibits several characteristics consistent with previous reports, such as a higher frequency in female patients (80.9%), ages ranging from 12 to 68 years (median 45.7 years), with xanthogranulomatous pyelonephritis frequently identified as the primary cause (47.6%), followed by pyonephrosis (38.1%) and renal infections (14.3%) (7). However, contrary to most cases which typically affect the left side (66.7%), our patient exhibited involvement on the right side (7).

Although there is evidence of success in conservative management, characterized by a positive response to antibiotic therapy and perfunctory drainage of the abscess, surgical resolution based on nephrectomy remains the mainstay even today (3, 9, 17–19). In the case of our patient, the treatment approach was in line with the literature and consisted of antibiotic therapy followed by nephrectomy. We consider that the long duration of the condition could have affected the results of antibiotic therapy (7, 19, 20).

Previous reports indicate that fatal outcomes in nephrobronchial fistulas are relatively uncommon and have been rare over the last two decades. However, the fatal outcome of nephrobronchial fistula is typically associated with acute respiratory failure due to purulent secretions through the endotracheal tube during surgery (21). In our patient’s case, the multiorgan failure observed postoperatively broadens the clinical perspective and highlights the serious complications that must be considered in the management of nephrobronchial fistulas, especially in cases with chronic progression.

The delay in the treatment of our patient, a frequent situation in developing countries, must be considered a crucial factor contributing to the patient’s fatal outcome, which underlines the critical importance of early treatment (17). In our patient, diagnosis was made secondary to the investigation of nonspecific symptoms, as described in previously reported cases (7). However, it should be noted that the onset of the patient’s respiratory symptoms was prolonged, leading to an unfavorable delay in accessing healthcare services, making the diagnosis an incidental finding, and contributing to delays in outpatient care. These characteristics, while determining the clinical course, also expose significant limitations in access to healthcare. Although the patient’s condition led to immediate in-hospital management with antibiotics and subsequent surgery, these actions ultimately proved insufficient.

This case underscores the necessity of considering a diagnosis of nephrobronchial fistula and pulmonary abscess, particularly when there is a history indicative of such conditions, as commonly seen in patients with this disease. Despite an imprecise clinical presentation, the presence of an ipsilateral thoracic lesion in a patient with renal disease (xanthogranulomatous pyelonephritis) should alert physicians to the potential for pulmonary extension of the renal process (7). A particularly timely, comprehensive approach, accompanied by a search for complications in unusual locations, is essential, because although this is a very rare complication, it can lead to high morbidity and mortality, as observed in our patient (7).

## 4 Conclusion

Nephrobronchial fistulas represent a very rare complication of renal infections, often presenting with nonspecific clinical symptoms. This case exemplifies the serious potential effects on morbidity and even mortality, especially in resource-limited settings where interventions may be delayed. It underscores the crucial importance of maintaining a high level of clinical suspicion and ensuring timely treatment.

## Data Availability

The original contributions presented in the study are included in the article/supplementary material, further inquiries can be directed to the corresponding author.
